# Letrozole and Ovarian Hyperstimulation Syndrome (OHSS): A Promising Prevention Strategy

**DOI:** 10.3390/jcm12020614

**Published:** 2023-01-12

**Authors:** Federica Di Guardo, Chiara Lello, Giosuè Giordano Incognito, Maria Teresa Bruno, Marco Palumbo

**Affiliations:** 1 Department of General Surgery and Medical Surgical Specialties, University of Catania, Via Santa Sofia 78, 95125 Catania, Italy; 2 Centre for Reproductive Medicine, Universitair Ziekenhuis Brussel, Vrije Universiteit Brussel, Laarbeeklaan, 101-1090 Brussels, Belgium; 3 Department of Drug and Health Sciences, University of Catania, 95125 Catania, Italy

**Keywords:** letrozole, ovarian hyperstimulation syndrome, ovarian stimulation

## Abstract

Ovarian Hyperstimulation Syndrome (OHSS) is an uncommon but serious complication occurring in patients undergoing ovarian stimulation. It is characterized by ovarian enlargement, nausea, vomiting, abdominal pain/distension, and reduction in urine output. However, OHSS may rarely evolve into a life-threatening condition with ascites, hemoconcentration and hypercoagulability. Prevention of OHSS consists of an integrated approach that associates behavioral aspects with administration of pharmacological compounds. Among drugs used to manage OHSS, Letrozole has recently been proposed as an effective option for prevention of the syndrome. However, despite the promising findings reported by several studies, to date Letrozole is not yet officially mentioned in the guidelines for “Prevention and Treatment of moderate and severe ovarian hyperstimulation syndrome”. In this scenario, the current study discusses Letrozole approaches scientifically available to prevent OHSS.

## 1. Introduction

Ovarian Hyperstimulation Syndrome (OHSS) is an uncommon but severe complication of assisted reproductive techniques (ART), determined by the use of gonadotrophins administered for controlled ovarian stimulation, under certain circumstances. However, the occurrence of OHSS is rare without the administration of human chorionic gonadotropin (hCG), which is used to trigger the ovulation. HCG is structurally and functionally similar to Luteinizing Hormone (LH), but with a longer half-life than LH [1,2]. The exposure of hyperstimulated ovaries to hCG is the key-event that promotes the synthesis of proinflammatory mediators. Among these, a variety of cytokines and angiogenic molecules, such as vascular endothelial grow factor (VEGF), are involved in the development of OHSS [3]. Uncontrolled ovarian enlargement associated with both local and systemic effects driven by proinflammatory mediators, including enhanced vascular permeability and a prothrombotic effect, are responsible for the clinical features of OHSS [4]. The symptoms of OHSS include mild to severe abdominal pain/distension until ascites associated with increased ovarian size, abdominal bloating, nausea, vomiting, diarrhea and reduction in urine output. Venous blood samples usually describe a status of hemoconcentration, hypercoagulability, and electrolyte alteration [5]. 

With regard to OHSS classification, the American Society for Reproductive Medicine (ASRM) distinguishes three categories: mild, moderate, or severe according to the entity of OHSS symptoms [6]. Based on the time of symptoms presentation from the trigger injection, OHSS can be defined as “early” and “late”, indicating a syndrome occurring in 9 days following the trigger injection or 10 or more days after, respectively [7,8]. 

However, it has to be mentioned that OHSS is a self-limiting condition, especially in patients who do not become pregnant, with resolution of symptoms at the time of the next menstrual period. Conversely, in patients who do conceive, the ovaries continue to be stimulated by the increasing hCG levels with symptoms that may last until the end of the first trimester. Moderate to severe OHSS occurs in roughly 1–5% of stimulated cycles [9,10,11,12,13]. Nevertheless, the real incidence is extremely difficult to estimate since a strict consensus on the definition is lacking.

Prevention of OHSS consists of an integrated approach that associates behavioral aspects with administration of pharmacological compounds.

## 2. Hypothesis

Among the drugs used to manage OHSS, Letrozole has recently been proposed as an effective option used for prevention of the syndrome [14,15]. Several hypotheses were proposed for the development of OHSS. An estrogen-mediated theory is explained by studies showing that patients with high serum estrogen levels during ovarian stimulation are at increased risk of OHSS [16]. Given this, Letrozole as nonsteroidal aromatase inhibitor, impedes the conversion of androgens into estrogens by blocking human aromatase in a potent, specific, and reversible way [17]. Administration of Letrozole during the luteal phase could also reduce the risk of thrombosis associated with this syndrome [15]. Moreover, OHSS is characterized by the excessive discharge of fluid from the blood vessels. After stimulated by hCG, granulosa lutein cells produce and release high levels of vascular endothelial growth factor (VEGF), which interacts with its receptor in the endothelial cells membrane and increases vascular permeability. A decrease in serum VEGF levels was also found after Letrozole administration [18]. Some authors supported the corpus luteum mediated hypothesis due to the luteotropic effect, with VEGF mediating downstream pathways [15,19]. Letrozole increases local androgen levels and thereby influences the granulosa lutein cells to decrease VEGF and E2 levels [15,20]. Patients receiving Letrozole have a shorter luteal phase and lower serum VEGF levels, indicating that the corpus luteum pathway is a plausible hypothesis. Thus, the use of Letrozole after oocyte retrieval may help lower estrogen and VEGF concentrations with the possible consequent reduction of OHSS incidence, opening a new scenario in the management of this syndrome. In this context, the presented study proposes the use of Letrozole as a medical prevention approach for OHSS.

## 3. Discussions

The clinical goal of ART is the birth of a healthy baby achieved after treatments such as ovarian stimulation, during which it is important to minimize the occurrence of potential risks for the mother. As OHSS is a rare but important complication of ovarian stimulation, expedients and therapies aimed to avoid and/or reduce its incidence have been investigated over the decades. In 2008, Fatemi reported that estrogen levels were significantly decreased after 5.0 mg Letrozole administration during luteal phase compared with placebo group [14]. In 2009, Garcia-Velasco reported that 2.5 mg of Letrozole can significantly decreased estrogens during luteal phase, which was consistent with the pharmacological mechanism of Letrozole, and proposed that it could be used to prevent the OHSS [15]. In the last few years, several clinical trials supported the efficacy of Letrozole in reducing the incidence of OHSS. Nevertheless, other studies did not replicate the same findings, showing that Letrozole is only able to reduce the estrogen levels without preventing the occurrence of the syndrome [21,22]. In this context, several authors questioned whether there is an efficient Letrozole dose able to reduce the incidence of OHSS and when it should be administered. Results of a study conducted in patients at high-risk for OHSS showed that doses of 2.5 mg, 5.0 mg, and 7.5 mg daily, orally administered for 5 consecutive days after oocyte retrieval, are able to decrease serum estrogen and VEGF levels. However, the higher dosage of 7.5 mg determined a significant reduction of OHSS incidence, in spite of the lower doses of 2.5 mg and 5 mg, which showed only a slight tendency in limiting the occurrence of OHSS [21]. Conversely, Wang et al. described that 5 mg of Letrozole daily for 5 days initiated during the luteal phase can significantly decrease serum estrogen levels on the 2nd, 5th, and 8th days after oocyte retrieval, but were not effective in reducing the incidence of severe OHSS [19]. These results were confirmed by the same team two years later [20]. On the other hand, it has to be mentioned that Letrozole has also been compared with other drugs, such as aspirin, for prevention of early OHSS. Mai et al. concluded that using 5 mg Letrozole daily for 5 days was more effective than low-dose aspirin in decreasing the incidence of moderate and severe early OHSS and was associated with higher VEGF levels. In addition, this study’s findings, described as pathogenesis of OHSS, might be caused by a luteolytic effect rather modulation of VEGF. Paradoxically, patients treated with Letrozole had significantly higher VEGF levels compared to the control group. The cause of increased VEGF attributed to aromatase action of Letrozole decreasing the estrogen serum concentration, resulting in disinhibition of the pituitary and increased gonadotropin stimulation of the ovaries to produce VEGF. They also found that a shorter luteal phase was seen in the Letrozole group compared to the control. The luteolytic hypothesis based on an observation that clinical symptoms of early OHSS disappear after the end of the luteal phase was used to explain the decrease in OHSS incidence in patients treated with Letrozole [22]. Similarly, Choudhary et al. showed that Letrozole and ganirelix acetate are equally effective for the overall prevention of OHSS, whereas Letrozole was more effective in preventing moderate OHSS. Moreover, Letrozole had better patient satisfaction and was less costly compared to GnRH antagonists [23].

It is important to mention that, to date, despite the presence of several studies about the use of Letrozole for OHSS prevention, this medication is still not mentioned in the guideline for “Prevention and Treatment of moderate and severe ovarian hyperstimulation syndrome” [6]. In addition, a recent systematic review and meta-analysis interestingly described that Letrozole could decrease the incidence of total OHSS as well as moderate and severe OHSS in high-risk women, while it seems to not be effective for the prevention of mild, moderate and severe OHSS, individually [24]. This is consistent with the results reported by Wang et al. [19,20]. However, the limitations of the study included the significant heterogeneity among the included studies’ characteristics, the small sample size and the lack of adjustment for meaningful confounders. In a recent study, a cocktail style treatment of GnRH-antagonist, mifepristone and Letrozole had enhanced synergistic effect on preventing the progression of OHSS in a rat model. [25]. Tshzmachyan et al., performed a randomized control trial investigating the effect of Letrozole on OHSS rates in high-risk polycystic ovary syndrome (PCOS) patients with elevated Anti-Mullerian Hormone (AMH) undergoing short GnRH therapy. They demonstrated that co-treatment with Letrozole during gonadotropin stimulation was able to significantly decrease the incidence of OHSS compared to the control group receiving standard short GnRH therapy protocol and had reduced serum estrogen levels [26].

In conclusion, although the use of Letrozole for OHSS prevention has not yet gained “official” acceptance, promising findings seem to support its administration as an effective therapeutical option to reduce OHSS incidence. The oral administration of 7.5 mg Letrozole daily for 5 consecutive days beginning on the day of oocyte retrieval seems to be the best option to prevent OHSS in high-risk women. In the future, large prospective randomized trials are required to evaluate the effect of Letrozole and its endocrine impact on the development of OHSS. 

## Data Availability

Not applicable.
